# Bacterial Outer Membrane Vesicle (OMV)-Encapsulated TiO_2_ Nanoparticles: A Dual-Action Strategy for Enhanced Radiotherapy and Immunomodulation in Oral Cancer Treatment

**DOI:** 10.3390/nano14242045

**Published:** 2024-12-20

**Authors:** Shun-An Kan, Li-Wen Zhang, Yu-Chi Wang, Cheng-Yu Chiang, Mei-Hsiu Chen, Shih-Hao Huang, Ming-Hong Chen, Tse-Ying Liu

**Affiliations:** 1 Department of Medical Education, Taipei Veterans General Hospital, Taipei 112, Taiwan; sakan@vghtpe.gov.tw; 2 Department of Biomedical Engineering, National Yang Ming Chiao Tung University, Taipei 112, Taiwan; lwzhang@eirgenix.com (L.-W.Z.); tomato13590.be04@nycu.edu.tw (Y.-C.W.); chiangjim03.be11@nycu.edu.tw (C.-Y.C.); 3 Department of Internal Medicine, Far Eastern Memorial Hospital, New Taipei 220, Taiwan; femh61665@femh.org.tw; 4 Division of Neurosurgery, Department of Surgery, Far Eastern Memorial Hospital, New Taipei 220, Taiwan; femh87905@femh.org.tw; 5 Department of Healthcare Administration, Asia Eastern University of Science and Technology, New Taipei 220, Taiwan; 6 Department of Electrical Engineering, Yuan Ze University, Taoyuan 320, Taiwan

**Keywords:** oral cancer, TiO_2_ nanoparticles, radiotherapy, outer membrane vesicles

## Abstract

Oral squamous-cell carcinoma (OSCC) poses significant treatment challenges due to its high recurrence rates and the limitations of current therapies. Titanium dioxide (TiO_2_) nanoparticles are promising radiosensitizers, while bacterial outer membrane vesicles (OMVs) are known for their immunomodulatory properties. This study investigates the potential of OMV-encapsulated TiO_2_ nanoparticles (TiO_2_@OMV) to combine these effects for improved OSCC treatment. TiO_2_ nanoparticles were synthesized using a hydrothermal method and encapsulated within OMVs derived from Escherichia coli. The TiO_2_@OMV carriers were evaluated for their ability to enhance radiosensitivity and stimulate immune responses in OSCC cell lines. Reactive oxygen species (ROS) production, macrophage recruitment, and selective cytotoxicity toward cancer cells were assessed. TiO_2_@OMV demonstrated significant radiosensitization and immune activation compared to unencapsulated TiO_2_ nanoparticles. The system selectively induced cytotoxicity in OSCC cells, sparing normal cells, and enhanced ROS generation and macrophage-mediated antitumor responses. This study highlights TiO_2_@OMV as a dual-action therapeutic platform that synergizes radiotherapy and immunomodulation, offering a targeted and effective strategy for OSCC treatment. The approach could improve therapeutic outcomes and reduce the adverse effects associated with conventional therapies.

## 1. Introduction

Oral cancer encompasses various malignant tumors that occur within the oral cavity, with approximately 90% being squamous-cell carcinoma (SCC) [1]. These tumors can develop in regions such as the tongue, pharynx, floor of the mouth, buccal mucosa, gums, lips, palate, pharyngeal area, and salivary glands. According to the World Health Organization, an estimated 657,000 new cases of oral and oropharyngeal cancers are diagnosed annually, resulting in over 330,000 deaths. Despite advancements in treatment, the 5-year survival rate for oral squamous-cell carcinoma (OSCC) from 2009 to 2019 remains around 48% [2,3].

Current clinical treatments for oral cancer predominantly involve surgery and radiotherapy. However, the anatomical location of oral cancers, often within the facial region, means that surgical removal can have significant cosmetic and functional implications [4]. Furthermore, incomplete resection, lymph node metastasis, and extranodal extension often lead to high recurrence rates. Other treatment options, such as chemotherapy combined with targeted therapy, have limited efficacy due to the development of drug resistance in tumor cells, offering only a modest extension of patient survival by three to six months, alongside considerable side effects [5,6]. Radiotherapy, typically administered at total doses ranging from 50 to 70 Gy, is associated with severe complications, such as osteoradionecrosis and mucosal ulceration, affecting 40–70% of patients [7,8], while those complications could be decreased with the usage of radio-enhancers [9]. Given these challenges, there is a growing interest in developing immunotherapy as a novel treatment approach, while strategies to enhance antitumor immune responses by combining immunotherapy with traditional treatments, such as surgery, chemotherapy, and radiotherapy, are being explored for potential synergistic effects.

This study proposes using titanium dioxide (TiO_2_) nanoparticles combined with bacterial outer membrane vesicles (OMVs) as a novel delivery system for low-dose radiotherapy, aiming to achieve radiosensitization and immune response activation [10]. TiO_2_ nanoparticles, widely used as photocatalysts in industry, have shown promising anticancer effects by generating reactive oxygen species (ROS) under light, ultrasound, or radiation, leading to cancer cell membrane, mitochondrial, and DNA damage [11,12,13]. However, aqueous-phase TiO_2_ nanoparticles exhibit high toxicity to normal cells and tend to aggregate, limiting their efficacy [14,15,16]. To address these issues, this study synthesizes oil-phase TiO_2_ nanoparticles and encapsulates them with OMVs derived from Gram-negative bacteria [17,18,19]. OMVs, which carry bacterial antigens, can stimulate innate immune responses and have been used in vaccine development. Functionalized OMVs have demonstrated antitumor effects in several studies [20,21].

In summary, this research aims to enhance the radiosensitization effect and induce immune-related responses by using OMV-encapsulated oil-phase TiO_2_ nanoparticles. This approach is expected to selectively target cancer cells while minimizing toxicity to normal cells, improving therapeutic outcomes. Ultimately, this novel strategy seeks to offer clinicians and patients more treatment options, improving the quality of life and potentially extending the survival of oral cancer patients.

## 2. Materials and Methods

### 2.1. Experimental Design

In this study, titanium dioxide nanoparticles (TiO_2_ NPs) known for their radiosensitivity were encapsulated within a double-layer phospholipid structure derived from bacterial outer membrane vesicles (OMVs) isolated from Gram-negative bacteria. The TiO_2_@OMV carrier, in combination with radiation therapy, was designed to enhance reactive oxygen species (ROS) levels upon exposure to low-dose X-ray irradiation. Additionally, OMVs, which possess immunogenic properties, were expected to recruit macrophages to induce an antitumor immune response. The encapsulation of TiO_2_ NPs within OMVs aimed to facilitate the phagocytosis by oral cancer cells, extend the circulation time of the carrier in vivo, and address the hydrophobicity of oil-phase TiO_2_ NPs.

### 2.2. Synthesis of Materials

#### 2.2.1. Synthesis of Titanium Dioxide Nanoparticles (TiO_2_ NPs)

Titanium (IV) butoxide (5 mmol), oleic acid (30 mmol), oleylamine (20 mmol), and ethanol (100 mmol) were mixed in a 50 mL three-necked flask and heated to 100 °C. The heated mixture was then transferred to a hydrothermal autoclave and further heated to 185 °C for 17 h. Upon completion, the reaction product was washed multiple times with ethanol to remove unreacted substances, redissolved in pentane, and stored at 4 °C.

#### 2.2.2. Bacterial Culture and Outer Membrane Vesicle (OMV) Extraction

Escherichia coli (MAX Efficiency^TM^ DH5*α* Competent Cells, Thermo Fisher, Waltham, MA, USA), were cultured in lysogeny broth (1% tryptone, 0.5% yeast extract, 1% NaCl, pH 7.0) at 37 °C with shaking at 180 rpm for 16–18 h. The culture was then expanded 100-fold in fresh LB broth and incubated for an additional 16–18 h until the OD600 reached 0.8–1.0. Bacteria were pelleted by centrifugation at 5000 rpm for 20 min, and the supernatant was filtered through a 0.45 µm vacuum filter. The OMVs were concentrated using a centrifugal filter unit with a 100 kDa molecular-weight cutoff (Amicon, Millipore, Kenilworth, NJ, USA) and then further purified by centrifugation at 36,000 rpm for 3 h at 4 °C using an SW 41 Ti rotor (Beckman Coulter Inc., Brea, CA, USA). The OMV pellet was resuspended in PBS and stored at −80 °C [22].

#### 2.2.3. Synthesis of TiO_2_@OMV Nanoparticles

After protein quantification of the OMVs, the required concentration was diluted 3–5 times with PBS, ensuring that the aqueous phase volume was at least 10 times larger than the oil phase. TiO_2_ NPs (7 µg/µL) were added to the mixture and subjected to high-frequency ultrasonication in an ice bath for 1 min. The volume of TiO_2_ NPs used was calculated as 0.23 times the OMV protein concentration. The resulting TiO_2_@OMV suspension was then subjected to vacuum evaporation to remove the oil-phase solvent and stored at 4 °C.

### 2.3. Characterization of Materials

#### 2.3.1. Transmission Electron Microscopy (TEM)

The prepared samples were dispersed in sterilized water, diluted to an appropriate concentration, and 5 µL of each sample was placed onto a 200-mesh carbon-coated copper grid. Excess liquid was removed with filter paper, and the grid was dried at 60 °C overnight. Prior to imaging, the sample was further dried under vacuum. Images were captured using TEM (JEOL, JEM-2000EX II, Tokyo, Japan) at 200 kV.

#### 2.3.2. Protein Quantification

Protein concentration was determined using the Bradford protein-binding assay. Bovine serum albumin (BSA) was used to generate a standard curve. Various concentrations of BSA (0–2 mg/mL) were mixed with Bradford reagent and incubated at room temperature for 5 min. Absorbance was measured at 595 nm using a TECAN Sunrise ELISA Reader (TECAN, Zurich, Switzerland). The protein concentration of the samples was calculated based on the standard curve.

### 2.4. In Vitro Cell Experiments

#### 2.4.1. Cell Culture

Human tongue squamous-cell carcinoma cells (CAL27) were purchased from ATCC and cultured in Dulbecco’s modified Eagle’s medium (DMEM) supplemented with 10% FBS and 4 mM glutamine at 37 °C and 5% CO_2_. Cells were subcultured every 2–3 days.

Murine fibroblast cells (L929) were also purchased from ATCC and cultured in Minimum Essential Medium (MEM) supplemented with 10% FBS and 4 mM glutamine at 37 °C and 5% CO_2_. Cells were subcultured every 2–3 days without trypsinization.

Murine macrophage cells (RAW264.7) were purchased from BCRC and cultured in Dulbecco’s MEM supplemented with 10% FBS and 4 mM glutamine at 37 °C and 5% CO_2_. Cells were subcultured every 2–3 days without trypsinization.

#### 2.4.2. Cytotoxicity Assay

The cytotoxicity of OMVs and TiO_2_@OMV was assessed using PrestoBlue^®^ Cell Viability Reagent (Thermo Fisher, Waltham, MA, USA). The cells (2 × 10^4^) were seeded in 24-well plates, and after 24 h, they were treated with varying concentrations of OMVs or TiO_2_@OMV (0–10 µg/mL) for 24 h. The cells were then washed with PBS, and PrestoBlue reagent diluted 20 times and serum-free medium was added. After 20 min of incubation at 37 °C, 100 µL of the supernatant was transferred to a black plate, and fluorescence was measured at 560/590 nm using a TECAN Sunrise ELISA Reader.

#### 2.4.3. Cell Viability Post-Radiation Treatment

Cells (2 × 10^4^) were seeded in 24-well plates and treated with varying concentrations of TiO_2_@OMV (0–10 µg/mL) for 12 h. The cells were then exposed to X-ray radiation (6 MeV, dose rate of 100 rad/min, total dose of 2 Gy) and returned to the incubator for 24 h. Cell viability was assessed as described above at 24, 48, and 72 h post radiation treatment.

#### 2.4.4. Co-Culture with Macrophages Post Radiation Treatment

CAL27 cells (2 × 10^4^) were seeded in 24-well plates and treated with OMVs or TiO_2_@OMV (5 µg/mL) for 12 h. The cells were then exposed to X-ray radiation and immediately co-cultured with RAW264.7 macrophages (2 × 10^5^) in a transwell insert. After 48 h, cell viability was assessed using PrestoBlue reagent as described above.

### 2.5. Molecular Biology Experiments

#### 2.5.1. Cell Cycle Analysis Pre Radiation

CAL27 and L929 cells (4 × 10^5^) were seeded in 6-well plates and treated with TiO_2_@OMV (0–10 µg/mL) for 24 h. The cells were harvested, washed with PBS, and fixed in 70% ethanol at −20 °C overnight. Fixed cells were stained with PI/Triton X-100 solution and analyzed using flow cytometry.

#### 2.5.2. Intracellular ROS Detection

CAL27 cells were seeded at a density of 4 × 10^5^ cells per well in a 6-well plate. The following day, after the cells adhered, they were washed with PBS. Subsequently, 5 µg/mL of OMV and 5 µg/mL of TiO_2_@OMV were added and incubated for 12 h. After incubation, the cells were washed again with PBS, detached using Trypsin, and transferred to an Eppendorf tube. The cells were then centrifuged at 1000 rpm for 5 min to remove the supernatant. Next, 100 µL of culture medium and 100 µL of DCFDA (2′,7′-Dichlorofluorescin diacetate, 20 µM) were added, and the mixture was incubated for 30 min. Following the incubation, the cells were exposed to X-ray irradiation (6 MeV, with a dose rate of 100 rad/min, and a total dose of 2 Gy). After irradiation, 100 µL of the cell suspension was transferred to a black plate, and fluorescence was measured using a TECAN Sunrise ELISA Reader with excitation/emission set at 485/535 nm.

#### 2.5.3. Fluorescent Staining for Carrier Phagocytosis

CAL27 and L929 cells were seeded at a density of 6 × 10^4^ cells per well in a 12-well plate containing 18 mm round coverslips. After allowing the cells to adhere overnight, they were washed with PBS, and 10 µg/mL of TiO_2_@OMV was added to each well. The cells were incubated for 3, 12, or 24 h, then the supernatant was removed, and the cells were washed with PBS. The cells were fixed with 4% formalin for 15 min, followed by permeabilization with 0.1% Triton X-100 for 15 min. The cells were washed three times with PBS, and then 1 mL of Actin (Alexa Fluor 488, Thermo Fisher, Waltham, MA, USA), diluted 1:500 in PBS, was added to each well for 1 h. DAPI was added prior to coverslip mounting. The slides were then observed under a confocal microscope or stored at 4 °C.

#### 2.5.4. Fluorescent Staining for Immune Cell Migration

CAL27 cells were seeded at a density of 6 × 10^4^ cells per well in a 12-well plate containing 18 mm round coverslips. After allowing the cells to adhere overnight, they were washed with PBS, and 5 µg/mL of OMV and 5 µg/mL of TiO_2_@OMV were added to the wells and incubated for 12 h. After incubation, the cells were exposed to X-ray irradiation (6 MeV, with a dose rate of 100 rad/min, and a total dose of 2 Gy). Following irradiation, RAW264.7 immune cells were detached, centrifuged at 1000 rpm for 5 min, and washed with PBS. The cells were then resuspended in PBS and stained with Vybrant™ DiD Cell-Labeling Solution, adding 5 µL of dye per 1 × 10^6^ cells. The cells were gently mixed and incubated at 37 °C for 20 min. After staining, the cells were centrifuged at 1000 rpm for 5 min to remove the supernatant and then washed once with PBS before being resuspended in PBS. Finally, 6 × 10^5^ RAW264.7 cells were added to the 3 µm transwell insert and co-cultured for 24 h. The cells were then washed three times with PBS, fixed with 4% formalin for 15 min, washed three more times with PBS, stained with Actin (Alexa Fluor 488) for 30 min, and mounted with DAPI for observation under a confocal microscope.

#### 2.5.5. Western Blot Analysis

After treatment, the cells and RAW264.7 immune cells were co-cultured on a 0.4 µm transwell for 24 h. The cells were then scraped off, washed with PBS, and centrifuged at 4000 rpm for 4.5 min. The cell pellets were lysed with an appropriate amount of lysis buffer, followed by several freeze–thaw cycles between −80 °C and 37 °C. The samples were then placed in an ultrasonic bath for 15 min and centrifuged at 13,000 rpm for 15 min. The supernatant was collected for protein quantification. The proteins were separated by SDS-PAGE, transferred onto a PVDF membrane, and analyzed using primary and secondary antibodies. The final detection was performed using a Luminescence/Fluorescence Imaging System.

## 3. Results

### 3.1. Synthesis of Materials

#### 3.1.1. Morphology of Oil-Phase Titanium Dioxide Nanoparticles

**Figure 1:**
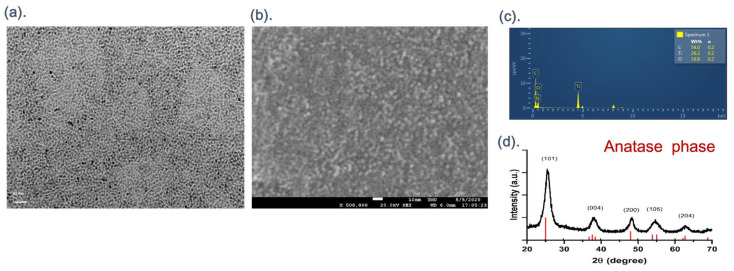
(**a**) TEM images of the TiO_2_ NPs synthesized by TB/OA/OM = 1:6:4 molar ratio, the formation of spherical particles of TiO_2_ NPs with an average size of 2–5 nm. (**b**) SEM images of the TiO_2_ NPs with an aggregated spherical structure. (**c**) EDX of the TiO_2_ NPs, showing the composition of carbon (C), oxygen (O), and titanium (Ti) elements. (**d**) XRD of the TiO_2_ NPs.

Titanium dioxide (TiO_2_) nanoparticles were successfully synthesized using the hydrothermal method. Transmission electron microscopy (TEM) images at a magnification of 5 million times (Figure 1a) revealed that the TiO_2_ nanoparticles were uniform in size and exhibited a spherical structure, with measured diameters ranging from 2 to 5 nm. Scanning electron microscopy (SEM) at a magnification of 500,000 times (Figure 1b) confirmed that the TiO_2_ nanoparticles formed an aggregated spherical structure. Energy-dispersive X-ray spectroscopy (EDX) analysis performed during SEM imaging (Figure 1c) identified the nanoparticles as being composed of carbon (C), oxygen (O), and titanium (Ti) elements. X-ray diffraction (XRD) analysis (Figure 1d), conducted over an angle range of 20–70 degrees, showed diffraction peaks corresponding to the (101), (004), (200), (105), and (204) planes, consistent with the anatase phase of TiO_2_.

#### 3.1.2. Morphology of Bacterial Outer Membrane Vesicles (OMVs) Coated with Titanium Dioxide Nanoparticles

**Figure 2:**
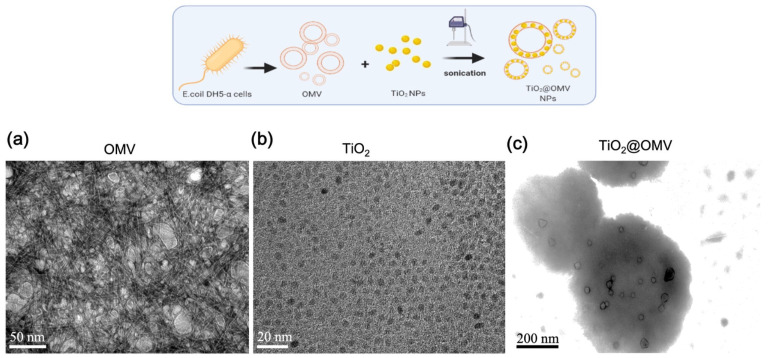
Preparation and characterization of DH5-α outer membrane-coated nanoparticles (TiO_2_@OMV NPs). TEM images of (**a**) OMV NPs, (**b**) bare TiO_2_, and (**c**) TiO_2_@OMV NPs. (**a**,**c**) Samples were negatively stained with phosphotungstic acid before imaging.

In this study, titanium dioxide (TiO_2_) nanoparticles were encapsulated within bacterial outer membrane vesicles (OMVs) using high-frequency ultrasonication. Transmission electron microscopy (TEM) images at a magnification of 10 million times were used to observe the structures. As shown in Figure 2a, the OMVs, after negative staining with phosphotungstic acid, exhibited a reverse contrast appearance, with their sizes varying between 10 and 200 nm. Figure 2b presents TEM images of TiO_2_ nanoparticles, measuring approximately 2–5 nm in diameter. The TEM image in Figure 2c displays the resulting structure after ultrasonication, a 200–400 nm spherical structure of OMV encapsulating TiO_2_, where the previously white phospholipid layer of the OMVs, post negative staining, turned black. This change in contrast suggests that the TiO_2_ nanoparticles were successfully integrated into the bilayer phospholipid membrane of the OMVs, causing the initially white layer to appear dark due to the densely packed nanoparticles.

### 3.2. Cytotoxicity Testing of OMVs

**Figure 3:**
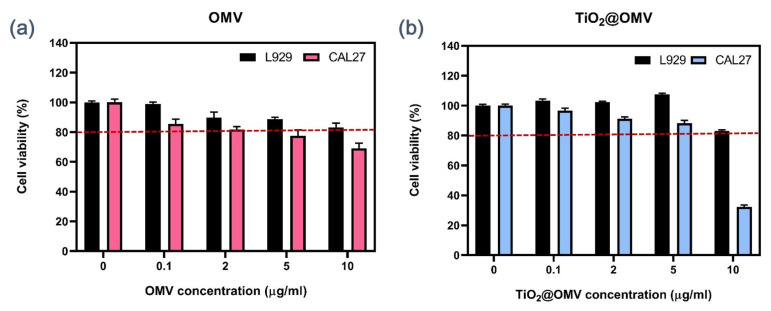
(**a**) Cell viability using PrestoBlue Cell Viability Reagent for CAL27 and L929 cells exposed to OMV at different concentrations (0, 0.1, 2, 5, 10 μg/mL) at 24 h. (**b**) Cell viability using PrestoBlue Cell Viability Reagent for CAL27 and L929 cells exposed to TiO_2_@OMV nanoparticles at different concentrations (0, 0.1, 2, 5,10 μg/mL) at 24 h. The results are expressed as mean ± standard deviation (SD) of n = 3 biologically independent samples. Statistical analysis was performed using two-way ANOVA.

Different concentrations of OMVs were mixed with serum-containing culture media and introduced to both normal L929 cells and CAL27 cancer cells, followed by a 24 h incubation to assess the cytotoxicity of the OMV carriers. As shown in Figure 3a, the results indicated a slight selectivity of OMVs between normal and cancerous cells. Even at a low concentration of 0.1 µg/mL OMVs, a differential effect was observed. At a concentration of 5 µg/mL, the survival rate of CAL27 cancer cells decreased to 80%, while the survival rate of normal L929 cells remained above 90%.

### 3.3. Cytotoxicity Testing of TiO_2_@OMV

To evaluate the cytotoxicity of TiO_2_@OMV carriers, varying concentrations of TiO_2_@OMV were mixed with serum-containing culture media and introduced to both normal L929 cells and CAL27 cancer cells, followed by a 24 h incubation. As depicted in Figure 3b, the results revealed a marked selectivity of TiO_2_@OMV carriers towards cancer cells at a concentration of 10 µg/mL. At this concentration, the survival rate of CAL27 cancer cells decreased significantly, while the survival rate of normal L929 cells remained above 80%, which could be considered indicative of a healthy and non-toxic environment [23,24].

### 3.4. Fluorescent Staining of Carrier Phagocytosis

**Figure 4:**
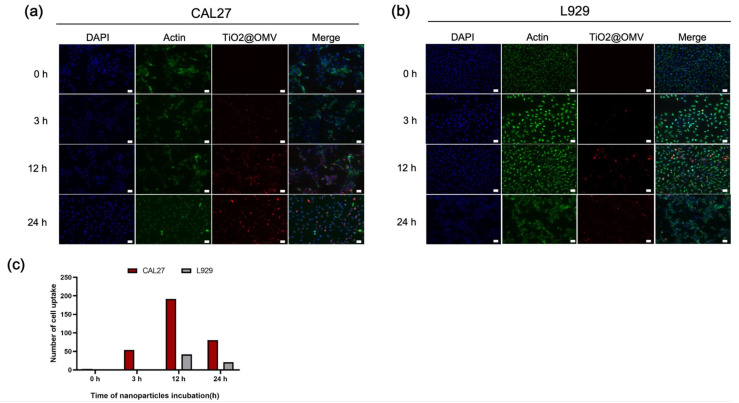
Fluorescence microscopic images of the localization of TiO_2_@OMV nanoparticle uptake in (**a**) CAL27 cells and (**b**) L929 cells treated with TiO_2_@OMV nanoparticle for 0, 3, 12, and 24 h. TiO_2_@OMV nanoparticle concentration: 10 μg/mL (TiO_2_@OMV NPs were labeled with vibrant DiD (red) fluorescence). Nucleuses were labeled with vibrant DAPI (blue) fluorescence. Actin was labeled with phalloidin (green) fluorescence. (**c**) The statistics of cell uptake with MetaMorphsoftware 7.10.4. Representative images were taken by a Leica fluorescence microscope at a magnification of 20× (Scale bar = 50 μm).

In this experiment, the TiO_2_@OMV carriers were mixed with serum-containing medium and subsequently introduced to CAL27 oral cancer cells and L929 normal cells to observe the phagocytosis at different time points. The red fluorescence indicates the localization of the TiO_2_@OMV carriers. As shown in Figure 4a, cancer cells exhibited the highest phagocytosis at 12 and 24 h. Similarly, Figure 4b demonstrates that L929 cells also showed significant phagocytosis at these time points. However, a comparative analysis reveals that the TiO_2_@OMV carriers were more extensively phagocytosed by cancer cells than by normal cells. This differential uptake is likely to influence the previously discussed cell survival rates.

### 3.5. Pre-Radiotherapy Cell Cycle Analysis of the Carrier

**Figure 5:**
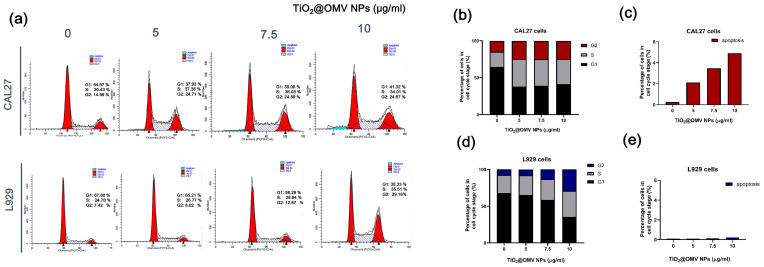
The effects of TiO_2_@OMV nanoparticles on cell cycle distribution and apoptosis. (**a**) CAL27 and L929 cells were treated with media containing 0, 5, 7.5, and 10 μg/mL TiO_2_@OMV nanoparticles. After 24 h, the cells were harvested and fixed in ice alcohol. Then, the cells were incubated in PBS containing 40 μg/mL propidium iodide and 100 μg/mL RNase A. Propidiumiodide-labeled nuclei were analyzed by flow cytometry. (**b**) The statistics of CAL27 cell cycle. (**c**) The statistics of CAL27 apoptosis. (**d**) The statistics of L929 cell cycle. (**e**) The statistics of L929 apoptosis.

The cell cycle is typically divided into the interphase (I phase) and mitotic phase (M phase). The interphase is the period of preparation and energy accumulation, while the mitotic phase is the process of cell proliferation. The entire cycle is represented as I phase → M phase. The interphase can be further subdivided into the G1 phase (gap 1), where DNA synthesis preparation occurs; the S phase (synthesis), where DNA synthesis takes place; and the G2 phase (gap 2), following DNA synthesis. During these stages, the main tasks are the replication of chromatin and the synthesis of associated proteins. With this subdivision, the entire cell cycle can be expressed as: G1 phase → S phase → G2 phase → M phase. Cells in the G2/M phase are notably more sensitive to radiation (Figure 5a).

As shown in the statistical results of Figure 5b, when CAL27 cancer cells were co-cultured with 5, 7.5, and 10 µg/mL of the carrier, the proportion of cells in the G2/M phase increased to twice that of the control group. In contrast, normal L929 cells showed a significant increase in the G2/M phase when co-cultured with 10 µg/mL of the carrier. However, these results are not ideal for the carrier design in this experiment. Although a concentration of 10 µg/mL demonstrated high cytotoxic selectivity towards CAL27 cancer cells, the effects on normal cells must also be considered when combined with radiation therapy. Therefore, 10 µg/mL is not the optimal concentration for this study. Instead, the 5 and 7.5 µg/mL concentrations are considered the best options. Comparing the statistical results of Figure 5c,e, it is evident that these concentrations cause significant apoptosis in cancer cells compared to normal cells.

### 3.6. Intracellular ROS Detection

**Figure 6:**
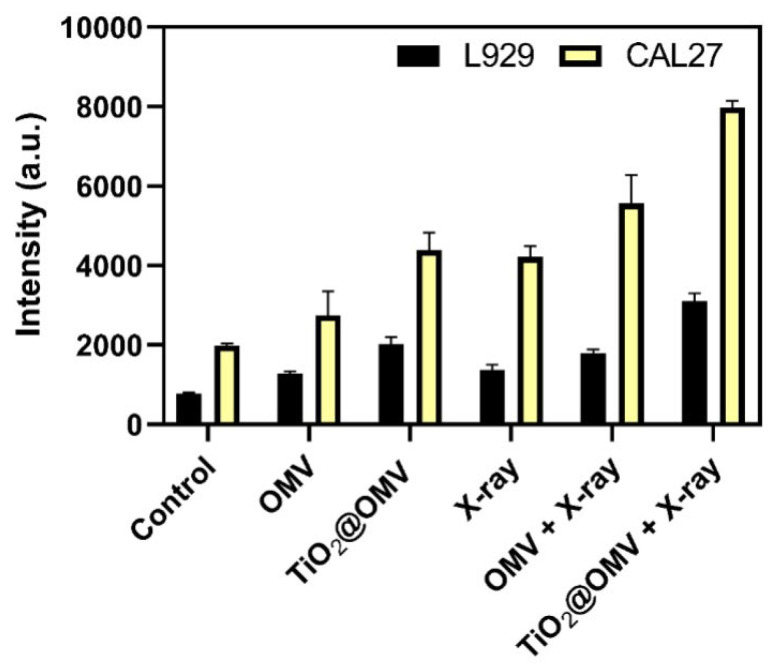
The generation of ROS from TiO_2_@OMV, detected by DCFDA after being exposed to a 6 MeV X-ray beam at radiation doses of 2 Gy (excitation: 485 nm and emission: 535 nm). The results are expressed as mean ± standard deviation (SD) of n = 3 biologically independent samples. Statistical analysis was performed using two-way ANOVA.

Reactive oxygen species (ROS) are byproducts of aerobic metabolism in biological systems, including oxygen ions, peroxides, and oxygen-containing free radicals. Due to their unpaired free electrons, ROS are highly reactive and can damage cellular and genetic structures when present at elevated levels. Consequently, many studies have exploited ROS to selectively kill cancer cells. As shown in Figure 6, the introduction of OMVs alone results in only a slight increase in ROS production, while X-ray irradiation alone generates more than double the amount of ROS. Notably, the ROS levels induced by the TiO_2_@OMV carrier combined with radiation therapy are significantly higher compared to the radiation-only group, indicating a synergistic effect in enhancing ROS production.

### 3.7. Cell Viability of CAL27 Cancer Cells Following TiO_2_@OMV Carrier-Mediated Radiation Therapy

**Figure 7:**
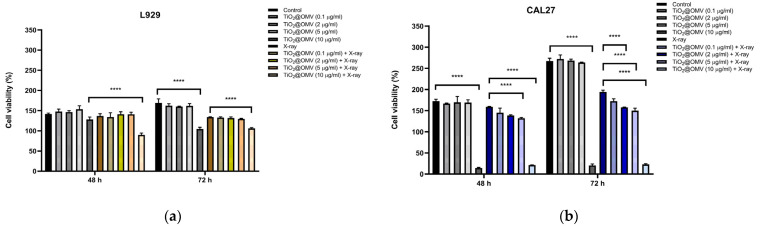
Cell viability of (**a**) CAL27 oral cancer cells, and (**b**) L929 normal cells incubated with TiO_2_@OMV nanoparticles (various concentrations = 0, 0.1, 2, 5, 10 μg/mL) for 12 h and subjected to X-ray irradiation (6 MV, 2 Gy) or no irradiation. The results are expressed as mean ± standard deviation (SD) of n = 3 biologically independent samples. Statistical analysis was performed using two-way ANOVA **** *p* < 0.0001).

This experiment investigated the potential radiosensitizing effect of TiO_2_@OMV carriers on CAL27 oral cancer cell by assessing cell viability at 48 and 72 h post radiation treatment. The results shown in Figure 7a demonstrate a significant decrease in cell viability at TiO_2_@OMV concentrations of 5 µg/mL and 10 µg/mL, suggesting enhanced radiosensitivity at these concentrations. Additionally, Figure 7b illustrates that L929 normal cells exhibited a notable decrease in viability at 10 µg/mL post radiation, while concentrations of 0.1, 2, and 5 µg/mL maintained cell viability above 90% at both 48 and 72 h. Based on these findings, a concentration of 5 µg/mL TiO_2_@OMV is considered optimal for maximizing radiosensitization while minimizing damage to normal cells.

### 3.8. Fluorescent Staining of Immune Cell Migration

**Figure 8:**
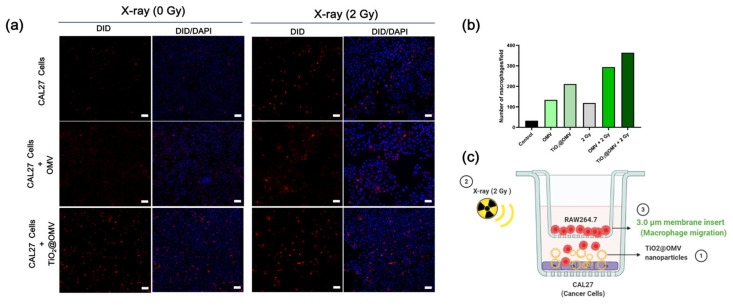
(**a**) Macrophage migration was analyzed 24 h after counterstaining cells in the lower chamber with DAPI. Representative images were taken by a Leica fluorescence microscope at a magnification of 20× (Scale bar = 50 μm). (**b**) The statistics of red fluorescent macrophages seeded to bottom chambers. (**c**) The presentation of the macrophage–cancer cell co-culture set up in transwell plates (the 3 μm microporous membrane allows for the migration of macrophages). DiD-labeled macrophages were seeded onto the insert and CAL27 cancer cells were seeded onto the bottom chamber. Cancer cells treated with OMVs (5 ug/mL) and TiO_2_@OMV (5 ug/mL) for 12 h and were subjected to X-ray irradiation (6 MV, 2 Gy) or no irradiation.

This experiment aimed to evaluate whether the designed carriers could attract immune cells to target cancer cells. A transwell system with 3 µm pores was employed, allowing macrophages to migrate through the membrane. Following treatment with the carriers and X-ray radiation, macrophages pre-labeled with Vybrant™ DiD Cell-Labeling Solution were placed in the transwell and cultured for 24 h. Using confocal microscopy, the migration of macrophages through the membrane was observed. The results showed that the combination of TiO_2_@OMV carriers with radiation treatment attracted the highest number of macrophages, indicating the potential for the enhanced immune cell targeting of cancer cells (Figure 8).

### 3.9. Survival Rate of Cancer Cells Co-Cultured with Macrophages After TiO_2_@OMV Carrier-Mediated Radiation Therapy

**Figure 9:**
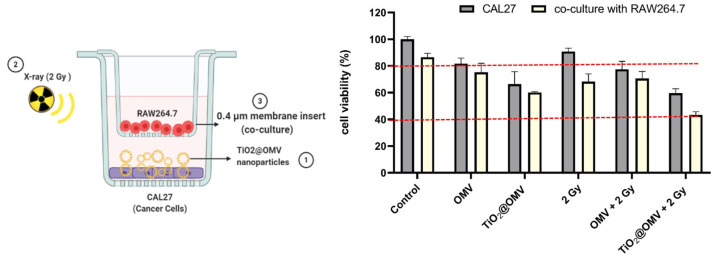
The cell viability of the macrophage–cancer cell co-culture set up in transwell plates (0.4 μm microporous membrane). Macrophages were seeded onto the insert and CAL27 cancer cells were seeded onto the bottom chamber. Cancer cells treated with OMVs (5 ug/mL) and TiO_2_@OMV (5 ug/mL) for 12 h and were subjected to X-ray irradiation (6 MV, 2 Gy) or no irradiation. The results are expressed as mean ± standard deviation (SD) of n = 3 biologically independent samples. Statistical analysis was performed using two-way ANOVA.

To evaluate the impact of macrophages on cancer cell viability following treatment with TiO_2_@OMV carriers and radiation, the cancer cells were co-cultured with macrophages after receiving the combined treatment. As shown in Figure 9, the presence of macrophages led to a 10% decrease in cancer cell survival in groups treated with OMVs, indicating that OMVs may enhance the ability of immune cells to suppress cancer cell growth. Notably, the group treated with both TiO_2_@OMV carriers and radiation showed the most significant reduction in cancer cell viability, with survival rates dropping to 40%. These results further confirm that this treatment strategy effectively attracts immune cells to the tumor site, where they subsequently exert antitumor effects. This promising outcome suggests potential for in vivo application.

### 3.10. Assessment of TNF-α and IL-6 Secretion in Macrophages Co-Cultured with TiO_2_@OMV Carriers Post Radiation Therapy

**Figure 10:**
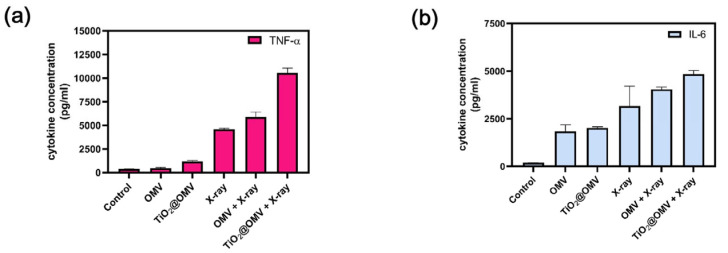
Cytokine levels of CAL27 cells medium. Macrophage–cancer cell co-culture and cancer cells treated with OMVs (5 ug/mL) and TiO_2_@OMV (5 ug/mL) for 12 h and were subjected to X-ray irradiation (6 MV, 2 Gy) or no irradiation. After two days, the ELISA kit analysis (**a**) of TNF-α Cytokine levels. (**b**) IL-6 Cytokine levels.

This study aimed to determine whether macrophages secrete tumor-associated factors, such as TNF-α and IL-6, when co-cultured with cancer cells treated with TiO_2_@OMV carriers and subjected to radiation therapy. Using ELISA kits, the secretion levels of these cytokines were measured and compared with the co-culture survival rates. The results indicated that both OMV and TiO_2_@OMV carriers significantly induced the production of TNF-α in the absence of radiation when compared to the control group. Notably, TiO_2_@OMV combined with radiation therapy led to the highest secretion of TNF-α, suggesting an enhanced immune response (Figure 10).

## 4. Discussion

Advancements in titanium dioxide (TiO_2_) nanoparticles have unveiled significant potential in enhancing cancer treatment modalities, particularly radiotherapy and photodynamic therapy (PDT). TiO_2_ nanoparticles have been established as effective radiosensitizers, primarily due to their ability to generate reactive oxygen species (ROS) under X-ray irradiation, which selectively amplifies DNA damage in cancer cells. This selective enhancement is crucial in minimizing collateral damage to surrounding healthy tissues, a persistent challenge in traditional radiotherapy. Moreover, the optimization of TiO_2_ nanostructures to improve their concentration and efficacy at tumor sites represents a promising step towards more targeted cancer therapies [25,26].

The selection of anatase-phase TiO_2_ as a radiosensitizer over rutile and brookite phases is primarily due to its superior photocatalytic efficiency and ability to generate reactive oxygen species (ROS) under UV irradiation. Anatase’s wider bandgap (~3.2 eV) and lower electron–hole recombination rate significantly enhance the production of hydroxyl radicals (•OH), which are crucial for inducing oxidative stress and DNA damage in cancer cells during radiotherapy [27]. Furthermore, anatase exhibits a greater surface area and reactivity compared to the rutile phase, which is known for its lower ROS generation due to its higher electron–hole recombination [28]. Brookite, though less explored, is less stable and more difficult to synthesize, limiting its practical use [29]. These factors make anatase the optimal choice for maximizing radiosensitizing effects while minimizing toxicity to normal tissues.

Beyond their role as radiosensitizers, TiO_2_ nanoparticles have also shown promise in multimodal therapies. When combined with chemotherapeutic agents like paclitaxel, TiO_2_ nanoparticles significantly enhance drug delivery, improving the bioavailability and therapeutic outcomes of these agents. Additionally, their role as photosensitizers in photodynamic therapy (PDT) and sonodynamic therapy (SDT) highlights their versatility, offering synergistic effects that improve the selective destruction of cancer cells. These findings underscore the potential of TiO_2_ nanoparticles as a multifunctional platform in cancer treatment [30].

Recent studies have also explored the application of TiO_2_ nanoparticles in immunotherapy. These nanoparticles can be engineered to deliver immunomodulators, thereby inducing a robust immune response against tumors. This approach has shown promise in activating key components of the immune system, such as dendritic cells and T cells, which play critical roles in targeting and destroying cancer cells. This dual functionality—enhancing both direct tumor destruction and immune-mediated tumor clearance—positions TiO_2_ nanoparticles as a compelling candidate for integrated cancer therapies [31].

In this study, we select hydrothermal synthesis in producing TiO_2_ nanoparticles, which was previously reported to show potential advantages in terms of cytotoxicity, particularly when targeting cancer cells. Research indicates that hydrothermal synthesis can yield highly crystalline TiO_2_ nanoparticles with controllable morphologies such as nanobelts, nanotubes, and nanorods. These specific structures are associated with increased surface area and enhanced photocatalytic activity, which can be harnessed for cancer treatments, including PDT. The high crystallinity and specific morphologies produced by this method can improve the generation of reactive oxygen species (ROS), which play a significant role in inducing cytotoxicity in cancer cells, while potentially sparing normal cells [32,33].

In parallel, bacterial outer membrane vesicles (OMVs) have gained attention for their ability to modulate immune responses and enhance antitumor immunity. OMVs, which can be engineered to carry tumor-associated antigens or immunomodulatory molecules, have shown promise as cancer vaccines. For instance, OMVs derived from Salmonella and functionalized with peptides like HPV E7 have effectively stimulated strong CD8+ T-cell responses, improving survival in preclinical models. This underscores the potential of OMVs as a platform for antigen-specific cancer vaccines [34].

OMVs are also being explored as carriers for chemotherapeutic agents, with functionalization enhancing their targeting and delivery capabilities. A notable example is the development of OMV-modified metal–organic frameworks (MOFs) for the treatment of triple-negative breast cancer. This innovative platform combines sonodynamic therapy with immunotherapy, leveraging OMVs to improve tumor targeting and stimulate immune activation, which has demonstrated significant tumor growth inhibition in preclinical models [31].

The enhanced cytotoxicity of Escherichia coli outer membrane vesicles (OMVs) toward cancer cells compared to normal cells, as observed in our study, may be attributed to several key factors. First, the structural components of OMVs, such as lipopolysaccharides (LPSs) and pore-forming proteins like hemolysin, allow for the selective targeting of cancer cells through enhanced permeability and retention (EPR) effects, which are prevalent in tumor microenvironments. This leads to the preferential accumulation of OMVs in cancer cells. Hemolysin, specifically, plays a crucial role by disrupting the cancer cell membrane, leading to mitochondrial dysfunction and the activation of apoptotic pathways, including caspase-3 and caspase-4, which are more sensitively triggered in cancer cells than in normal cells. Moreover, the altered metabolic and signaling pathways of cancer cells, along with their higher vulnerability to oxidative stress, make them more susceptible to the toxic effects of OMV-associated proteins [35,36].

The integration of outer membrane vesicles (OMVs) with nanoparticles has emerged as a powerful strategy in cancer therapy, combining the unique properties of both components to enhance therapeutic efficacy. OMVs, with their natural ability to target specific tissues and modulate immune responses, serve as highly effective carriers for nanoparticles. This combination has shown significant potential in improving the delivery and targeting of therapeutic agents to tumor sites. Notably, OMV-modified nanoparticles, such as metal–organic frameworks (MOFs), have demonstrated enhanced tumor targeting and therapeutic outcomes in preclinical cancer models, including breast cancer [31,37].

Furthermore, the synergistic effects observed in photodynamic and sonodynamic therapies highlight the potential of these hybrid systems. The functionalization of OMVs with nanoparticles enhances the delivery of photosensitizers, leading to improved reactive oxygen species (ROS) generation upon activation. This not only results in direct tumor cell cytotoxicity but also stimulates antitumor immune responses, providing a comprehensive approach to cancer treatment [26].

In the realm of immunotherapy and vaccine development, OMV–nanoparticle combinations offer promising avenues. By engineering OMVs to carry tumor antigens or immunostimulatory molecules, these platforms can potentiate the immune system’s response, leading to stronger and more durable antitumor immunity. This approach has significant implications for the development of cancer vaccines, potentially offering long-term protection against tumor recurrence [28,38].

The selective uptake of OMV-encapsulated TiO_2_ nanoparticles by cancer cells, as observed in our study, is strongly influenced by the heightened metabolic activity and increased endocytic capacity of tumor cells. Cancer cells, due to their rapid proliferation, require greater nutrient intake and exhibit enhanced endocytosis, which makes them more susceptible to nanoparticle uptake [39,40]. Furthermore, the tumor microenvironment, characterized by hypoxia and acidity, creates conditions that further enhance nanoparticle internalization, making cancer cells prime targets for treatments utilizing OMV-encapsulated nanoparticles [39].

According to the fluorescent staining of phagocytosis in our study, the cellular uptake of nanoparticles of both cancer and normal cells reaches its peak around 12 h, which likely represents the maximum efficiency of the endocytic pathways [40,41]. This observation is consistent with previous reports that describe rapid nanoparticle internalization in the early stages, followed by a plateau as the cell’s internalization machinery becomes saturated. The saturation point at 12 h may also reflect the balance between adsorption to the cell membrane and the desorption or exocytosis processes that limit further uptake [42]. The difference in cell uptake within normal cells and cancer cells could also be correlated with the difference in cell viability. Besides the difference in cell uptake, cancer cells are often more vulnerable to nanoparticle-induced cytotoxicity due to their compromised DNA repair mechanisms. Unlike normal cells, which have robust DNA repair systems, cancer cells frequently exhibit defective DNA repair pathways. This deficiency renders them more sensitive to DNA damage induced by reactive oxygen species (ROS) generated by TiO_2_ nanoparticles upon exposure to radiation [43]. The combination of high metabolic activity, increased nanoparticle uptake, and impaired DNA repair makes cancer cells particularly susceptible to the dual-action strategy employed by TiO_2_@OMV. This explains the observed higher sensitivity of cancer cells to TiO_2_@OMV-induced cytotoxicity in our study, as these cells are less equipped to manage the oxidative stress and DNA damage triggered by the treatment.

By leveraging the increased nanoparticle uptake and poor DNA repair capability of cancer cells, this approach ensures that the treatment is not only more effective at reducing tumor viability but also less toxic to normal cells. The enhanced selectivity and efficacy observed in our findings underscore the potential of OMV-encapsulated nanoparticles as a promising platform for targeted cancer therapy, offering a dual mechanism of radiosensitization and immune activation, while minimizing off-target effects [38,43].

Our findings also showed that TiO_2_ induces G2-/M-phase cell cycle arrest in cancer cells, aligning with observations in other cancer models [27]. For instance, studies on lung epithelial cells and breast cancer cells have shown that TiO_2_ nanoparticles cause G2/M arrest through mechanisms such as DNA damage and oxidative stress, or interference with key signaling pathways that regulate the cell cycle, such as the PI3K/AKT/mTOR pathway [44,45]. This effect is common across different cell types, as the nanoparticles interfere with crucial cell cycle checkpoints, preventing mitotic entry and promoting cytotoxicity.

The application of titanium dioxide (TiO_2_) nanoparticles and outer membrane vesicles (OMVs) in cancer therapy is promising, but several challenges need to be addressed for their successful clinical translation. For TiO_2_ nanoparticles, issues such as long-term toxicity, biocompatibility, and complex in vivo distribution remain significant hurdles. Upon systemic injection, TiO_2_ NPs can accumulate in non-target organs such as the liver, spleen, and lungs due to their high surface reactivity and prolonged retention times. This accumulation may lead to oxidative stress, inflammation, and tissue damage, especially in organs with active macrophage uptake. Studies have shown that excessive ROS production, while beneficial in cancer treatment, can cause oxidative damage to healthy cells, leading to systemic side effects. Long-term exposure to TiO_2_ NPs may also raise biocompatibility concerns. Their slow degradation rates can result in persistent nanoparticle retention, triggering chronic inflammatory responses or fibrosis in certain tissues.

Current efforts are focused on surface modifications, such as PEGylation or biomimetic coatings, to enhance stability and specificity, mitigating potential side effects. OMVs, while valuable for their immune modulation and targeted delivery capabilities, face challenges including the risk of systemic inflammatory responses and the need for precise engineering. Additionally, the combination of OMVs and nanoparticles presents further complexities, such as ensuring safety and controlling therapeutic agent release, requiring further optimization to expand their application across various cancer types. Future research will likely focus on overcoming these challenges to fully realize the therapeutic potential of these advanced materials [46,47].

In this study, we are the first to utilize the synergistic combination of titanium dioxide (TiO_2_) nanoparticles with bacterial outer membrane vesicles (OMVs) to develop a multifunctional therapeutic platform specifically designed for oral cancer treatment. Based on our assumptions about clinical application, our nanomaterials could be injected in situ as a future application. While TiO_2_ nanoparticles are well established as effective radiosensitizers due to their ability to generate reactive oxygen species (ROS), and OMVs have shown promise in targeted drug delivery and immune modulation, their integration in this study provides a novel approach that leverages the strengths of both components. Encapsulating TiO_2_ nanoparticles within OMVs not only enhances the targeted delivery and concentration of these nanoparticles at tumor sites but also utilizes the immunomodulatory properties of OMVs to attract and activate immune cells, thereby amplifying the antitumor immune response. This dual functionality addresses the limitations of traditional cancer therapies by improving tumor selectivity, minimizing off-target toxicity, and enhancing the immune system’s ability to target and destroy cancer cells. The differential effects observed in cytotoxicity and cell cycle analyses further demonstrate the potential of this combined approach to increase radiosensitivity in cancer cells while sparing normal cells, offering a promising new strategy for effective, targeted, and comprehensive cancer therapy.

There are still limitations in our study. Due to limitations in quantitative analysis techniques, we were unable to accurately determine the encapsulation efficiency of TiO_2_ within OMVs.

## 5. Conclusions

In this study, we developed an innovative carrier system for the treatment of oral cancer, utilizing TiO_2_@OMV as a delivery vehicle in combination with low-dose X-ray radiation. This approach aims to enhance reactive oxygen species (ROS) generation and leverage the immune-inducing properties of OMVs to recruit macrophages and initiate antitumor immune responses. Transmission electron microscopy (TEM) analysis confirmed the successful encapsulation of titanium dioxide within the OMV, as evidenced by the change in appearance of the phosphotungstic acid-stained phospholipid bilayer.

The TiO_2_@OMV carrier demonstrated significant radiosensitizing effects at a concentration of 5 µg/mL, with selective cytotoxicity towards oral cancer cells compared to normal cells. Confocal microscopy revealed a higher uptake of the carrier by CAL27 oral cancer cells after 12 h, and cell cycle analysis indicated an increase in the G2/M phase at concentrations of 5 and 7.5 µg/mL, suggesting optimal therapeutic efficacy at 5 µg/mL following 12 h of incubation prior to radiation treatment. Additionally, the results showed that the TiO_2_@OMV carrier effectively attracted macrophages to the tumor site and triggered a cascade of antitumor immune responses, leading to cancer cell death.

These preliminary findings suggest that the TiO_2_@OMV-based therapy holds promise as a novel treatment option for oral cancer, but still requires further in vivo validation.

## Data Availability

Data will be made available on request.
